# Identifying dementia outcomes in UK Biobank: a validation study of primary care, hospital admissions and mortality data

**DOI:** 10.1007/s10654-019-00499-1

**Published:** 2019-02-26

**Authors:** Tim Wilkinson, Christian Schnier, Kathryn Bush, Kristiina Rannikmäe, David E. Henshall, Chris Lerpiniere, Naomi E. Allen, Robin Flaig, Tom C. Russ, Deborah Bathgate, Suvankar Pal, John T. O’Brien, Cathie L. M. Sudlow, on behalf of Dementias Platform UK and UK Biobank

**Affiliations:** 1 Centre for Medical Informatics, Usher Institute of Population Health Sciences and Informatics, University of Edinburgh, Edinburgh, UK; 2 Centre for Clinical Brain Sciences, University of Edinburgh, Edinburgh, UK; 3 Anne Rowling Regenerative Neurology Clinic, University of Edinburgh, Edinburgh, UK; 4 Clinical Trial Service Unit and Epidemiological Studies Unit, Nuffield Department of Population Health, University of Oxford, Oxford, UK; 5 Alzheimer Scotland Dementia Research Centre, University of Edinburgh, Edinburgh, UK; 6 Centre for Dementia Prevention, University of Edinburgh, Edinburgh, UK; 7 James Cook University Hospital, Middlesbrough, UK; 8 Department of Psychiatry, University of Cambridge, Cambridge, UK

**Keywords:** Dementia, Alzheimer disease, Data accuracy, Predictive value of tests, Cohort studies, Validation studies

## Abstract

Prospective, population-based studies that recruit participants in mid-life are valuable resources for dementia research. Follow-up in these studies is often through linkage to routinely-collected healthcare datasets. We investigated the accuracy of these datasets for dementia case ascertainment in a validation study using data from UK Biobank—an open access, population-based study of > 500,000 adults aged 40–69 years at recruitment in 2006–2010. From 17,198 UK Biobank participants recruited in Edinburgh, we identified those with ≥ 1 dementia code in their linked primary care, hospital admissions or mortality data and compared their coded diagnoses to clinical expert adjudication of their full-text medical record. We calculated the positive predictive value (PPV, the proportion of cases identified that were true positives) for all-cause dementia, Alzheimer’s disease and vascular dementia for each dataset alone and in combination, and explored algorithmic code combinations to improve PPV. Among 120 participants, PPVs for all-cause dementia were 86.8%, 87.3% and 80.0% for primary care, hospital admissions and mortality data respectively and 82.5% across all datasets. We identified three algorithms that balanced a high PPV with reasonable case ascertainment. For Alzheimer’s disease, PPVs were 74.1% for primary care, 68.2% for hospital admissions, 50.0% for mortality data and 71.4% in combination. PPV for vascular dementia was 43.8% across all sources. UK routinely-collected healthcare data can be used to identify all-cause dementia in prospective studies. PPVs for Alzheimer’s disease and vascular dementia are lower. Further research is required to explore the geographic generalisability of these findings.

**Electronic supplementary material:**

The online version of this article (10.1007/s10654-019-00499-1) contains supplementary material, which is available to authorized users.

## Introduction

Dementia is a growing public health concern worldwide [1], and prospective, population-based studies are necessary to improve our understanding of its natural history and risk factors.

UK Biobank (UKB, www.ukbiobank.ac.uk) is a very large, prospective, population-based cohort study that was established to facilitate research into the determinants of health and disease, primarily in middle and old age [2]. UKB collected a wealth of exposure sociodemographic, lifestyle, environmental and health information during the baseline assessment, along with a range of physical measures and cognitive testing. Further enhancements include genotyping, repeat cognitive testing, dietary questionnaires and multimodal imaging. UKB is an open access resource, and any bona fide researcher around the world can apply to use its data for health-related research in the public interest. To date, UKB has approved projects to study dementia and cognitive disorders across a wide range of topics, including: identifying genetic, environmental and lifestyle risk factors for dementia; establishing the relationship between neuroimaging findings and cognition and developing dementia risk prediction models (www.ukbiobank.ac.uk/approved-research).

Follow-up for disease outcomes in UKB is largely via linkages to routinely-collected, coded clinical healthcare datasets [3]. UKB receives regularly updated linkages to national hospital admissions, cancer and mortality data for all participants, and has obtained linked primary care data for > 200,000 participants.

Attrition during follow-up can be a source of bias in longitudinal studies, and participants with poorer cognitive ability are at a greater risk of loss to active follow-up [4]. Passive follow-up using comprehensive data linkage minimises attrition and provides a cost-effective means of identifying disease cases in prospective studies.

These datasets must, however, identify cases with a high positive predictive value (PPV) (i.e., a high proportion of those with dementia codes in these datasets must be true dementia cases). Previous validation studies in the UK have investigated the PPV of single datasets, rather than in combination [5–8].

We aimed to estimate the PPV of dementia coding in UK primary care, hospital admissions and national mortality datasets alone and in combination using data from UKB.

## Methods

### Study design

We identified UK Biobank participants recruited in Edinburgh, Scotland, who had ≥ 1 dementia code in their linked UK hospital admissions, mortality or primary care data. We compared the coded diagnoses to diagnoses based on full-text electronic medical record (EMR) review by clinicians with dementia expertise as a reference standard.

### Recruitment to UK Biobank

Details regarding participant recruitment to UKB are published elsewhere [9, 10]. Briefly, between 2006 and 2010, UKB recruited 500,000 participants aged 40–69 years who were registered with the UK National Health Service (NHS) and living near one of 22 recruitment centres.

### Datasets and dementia codes

In the UK, mortality and hospital data are currently coded using the International Classification of Diseases version 10 (ICD-10) while primary care data are coded using the Read coding system (version 2 or 3). ICD-10 contains almost exclusively diagnostic codes, whereas the Read coding system includes diagnostic and administrative (e.g. specialist referral) codes (along with codes for prescriptions, procedures, symptoms and signs). We used a comprehensive four-stage process to compile a list of dementia ICD-10 and Read V2 codes (Online Resource 1), aimed at identifying cases with a high PPV, rather than at maximising sensitivity.

### Study population

We excluded participants with no correspondence in the local (National Health Service [NHS] Lothian) EMR system, as they are likely to obtain their healthcare in a different NHS area. We included all identified participants with any correspondence in the EMR (even if not pertaining to dementia) to avoid over-estimation of PPV due to information bias. The start date was the earliest code in any dataset and the end date was the latest date at which all three datasets were available (September 2015).

### Reference standard

The EMR contains hospital inpatient and outpatient correspondence as well as investigation results. To create case vignettes for adjudication, we extracted letters that referred to cognition or a diagnosis of dementia, along with any relevant neuroimaging and laboratory reports. We removed all personally identifying information from the vignettes. Using the case vignettes and a pre-piloted adjudication form (Online Resource 2), a clinician with dementia expertise (JO’B, SP, TR, DB or TW) determined whether dementia was present (‘all-cause dementia’) and, if so, whether a subtype diagnosis could be made. Diagnostic criteria were provided (Online Resource 3) [11–17]; however, since patients are frequently diagnosed with dementia in routine clinical practice without meeting rigorous formal criteria, the adjudicators could select a ‘formal criteria not met but diagnosis likely’ option, to indicate a diagnosis that they would make in their practice. We blinded researchers extracting the vignettes and adjudicators to the participants’ codes.

### Inter-rater agreement

Two clinicians independently adjudicated a random sub-sample of 25% of cases so that we could measure inter-rater agreement. We calculated the percentage agreement and Cohen’s kappa statistic for whether dementia was present or not and, where both adjudicators agreed dementia was present, the subtype diagnosis.

### Statistical analyses

For all-cause dementia, Alzheimer’s disease and vascular dementia, we calculated the PPV for each dataset separately, and for all three combined.

For all-cause dementia, true positive cases were those where the adjudicator recorded dementia as being present, with or without meeting diagnostic criteria. False positive cases were those where the adjudicator indicated that dementia was not present or where there was insufficient information to make a diagnosis of dementia. Adjudicating diagnoses for participants with insufficient information in their medical record to confirm or refute dementia may lead to an underestimate of PPV, so we also performed a sensitivity analyses in which we removed these participants from the PPV calculation for each dataset.

For dementia subtypes, cases were true positives if the adjudicator indicated that a particular subtype diagnosis could be made, with or without meeting the particular diagnostic criteria. False positive cases were those where it was not possible to determine the subtype diagnosis or if the adjudicator selected an alternative subtype diagnosis. We combined diagnoses of dementia with Lewy Bodies (DLB), Parkinson’s disease dementia (PDD) and frontotemporal dementia (FTD) into an ‘other specific dementias’ category due to small numbers for these diagnoses separately.

We calculated PPV as the number of true positives divided by the number of true and false positives combined, and calculated confidence intervals using the Clopper–Pearson (exact) method.

We investigated the effects on PPV and the numbers of cases ascertained by implementing additional criteria: using diagnostic versus administrative codes in primary care data; using subtype codes (such as Alzheimer’s disease or vascular dementia) to identify dementia of any cause; and requiring ≥ 2, ≥ 5 and ≥ 10 codes to identify all-cause dementia or Alzheimer’s disease. Based on these results, we identified algorithms that appeared to optimise a high PPV and good case ascertainment (implying reasonable sensitivity), as these are most likely to be of value to researchers using UKB data for dementia research.

We compared demographic data for false positives and true positives for all-cause dementia (age at recruitment, sex, age at first code, number of codes, whether participants died during follow-up and socioeconomic status as measured by the Townsend Deprivation Index [TDI]). The TDI was divided into quintiles, ranging from 1 (lowest deprivation) to 5 (highest deprivation), based on 2001 census data [18]. We used Microsoft SQL 2012 for data management and conducted statistical analyses in R (www.r-project.org).

## Results

### Participant characteristics

**Fig. 1:**
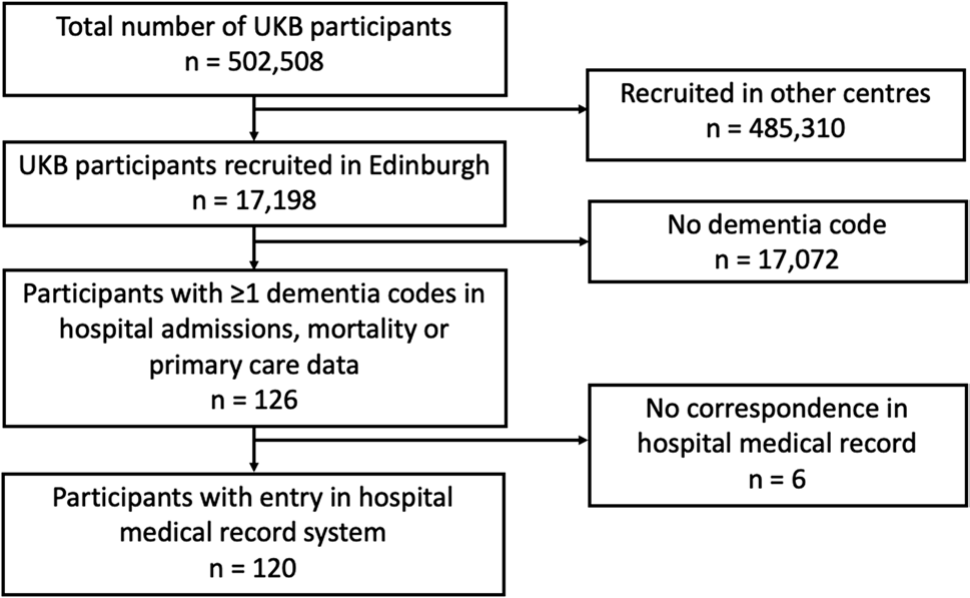
Flow diagram of participant selection

Of the 17,198 UKB participants recruited in Edinburgh, 126 had ≥ 1 dementia code in any dataset. Of these, there were no free-text entries in the medical record system for six participants, leaving 120 participants in the study (Fig. 1).

Of the 120 included participants, 64 (53.3%) were female, median age of recruitment was 67 years (range 43–70 years) and median age at receiving first dementia code was 70 years (range 41–77 years). Twenty-five participants (20.8%) died during follow-up.

**Fig. 2:**
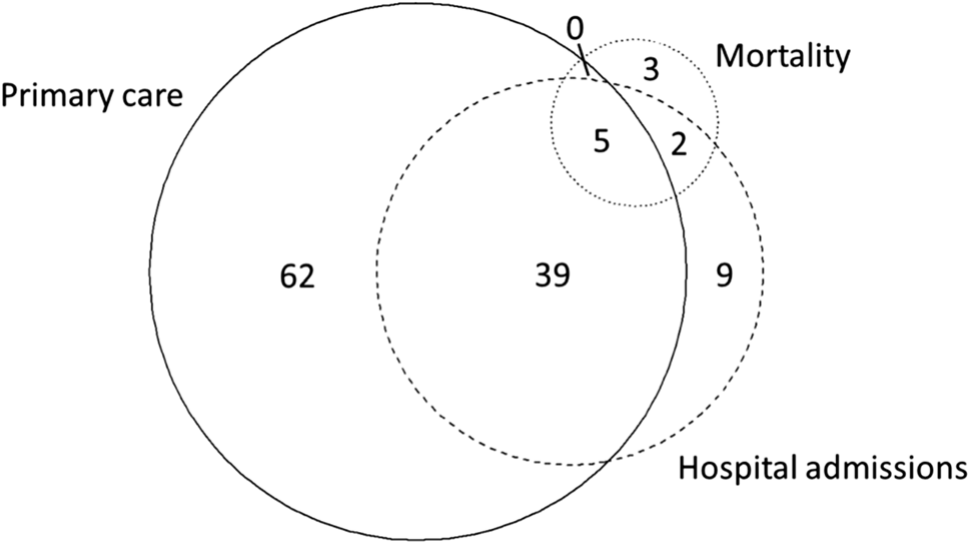
Area proportional Euler diagram indicating the datasets from which dementia cases were identified (n = 120). Distribution of cases identified at any time until end of follow-up (September 2015)

Across the 120 participants, there were 633 dementia codes in total, with a median of four codes per person (range 1–35). Of 389 primary care codes, 168 (43.2%) were diagnostic and 221 (56.8%) were administrative. The first dementia code was in primary care data in 79.2% of participants, hospital admissions data in 18.3% and mortality data in 2.5%. Of the 120 identified cases, 62 (51.7%) were found only in primary care data. The distribution of identified cases across the three datasets is displayed in Fig. 2.

### Reference standard diagnoses

**Fig. 3:**
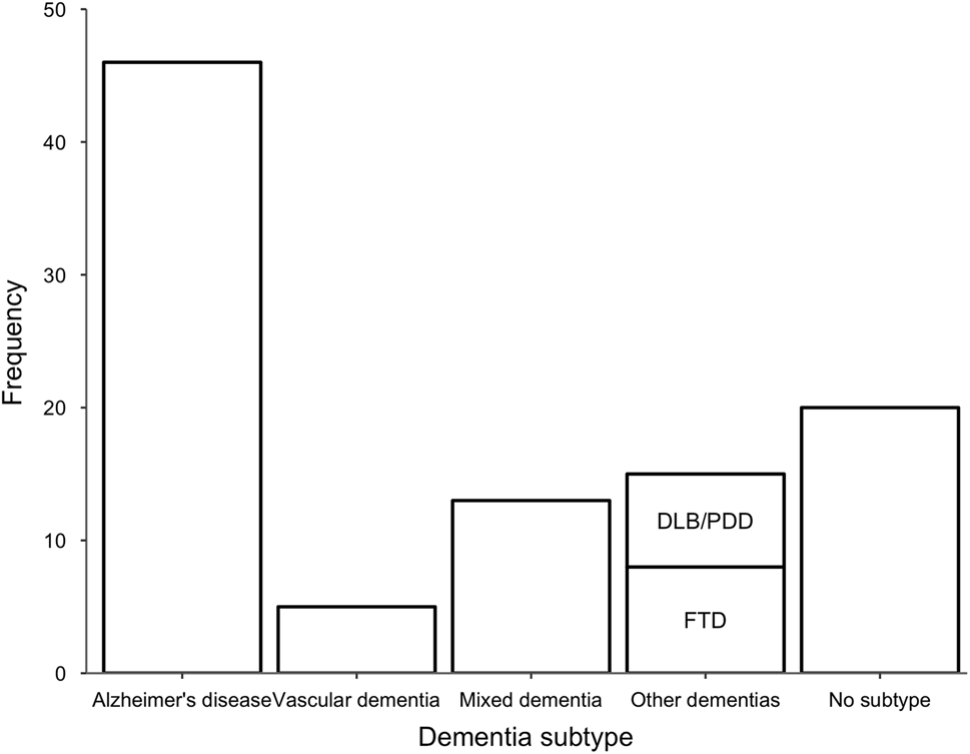
Adjudicator subtype diagnoses of the 99 cases adjudicated to have dementia. *AD* Alzheimer’s disease, *VaD* vascular dementia, *Mixed* mixed AD/VaD, *DLB/PDD* dementia with Lewy bodies and Parkinson’s disease dementia, *FTD* frontotemporal dementia

Adjudicators determined that dementia of any cause was present in 99/120 participants. Of these, 77 (77.8%) met the formal ICD-10 diagnostic criteria for dementia. Adjudicators were able to make a subtype diagnosis in 79/99 (79.8%) of cases (Fig. 3), with 38/79 (48.1%) meeting formal subtype diagnostic criteria. Alternative diagnoses for the cases judged not to have dementia were: mild cognitive impairment (9), affective disorders (2), delirium (1), stroke (1), unclear due to diagnostic uncertainty (2). Adjudicators deemed six further participants as not having dementia due to lack of relevant correspondence. In these cases, there was no correspondence in the medical record that mentioned cognitive symptoms, a diagnosis of dementia, or a relevant alternative diagnosis.

### Inter-rater agreement

Two adjudicators independently reviewed 30/120 (25%) of cases. The percentage agreement and kappa coefficients for all-cause dementia and dementia subtypes are displayed in Table 1.

**Table 1:** Adjudicator agreement

Agreement	Number agreed/total number	Percentage agreement (%)	Kappa coefficient (95% CI)
All-cause dementia	27/30	90	0.76 (0.48–1.00)
Dementia subtypes^a^	13/20	65	0.57 (0.29–0.84)

Percentage agreement and kappa coefficients for whether adjudicators agreed on the presence or absence of dementia or the particular subtype

^a^Among 20 cases where both adjudicators thought that all-cause dementia was present

### PPV for all-cause dementia, Alzheimer’s disease and vascular dementia

**Fig. 4:**
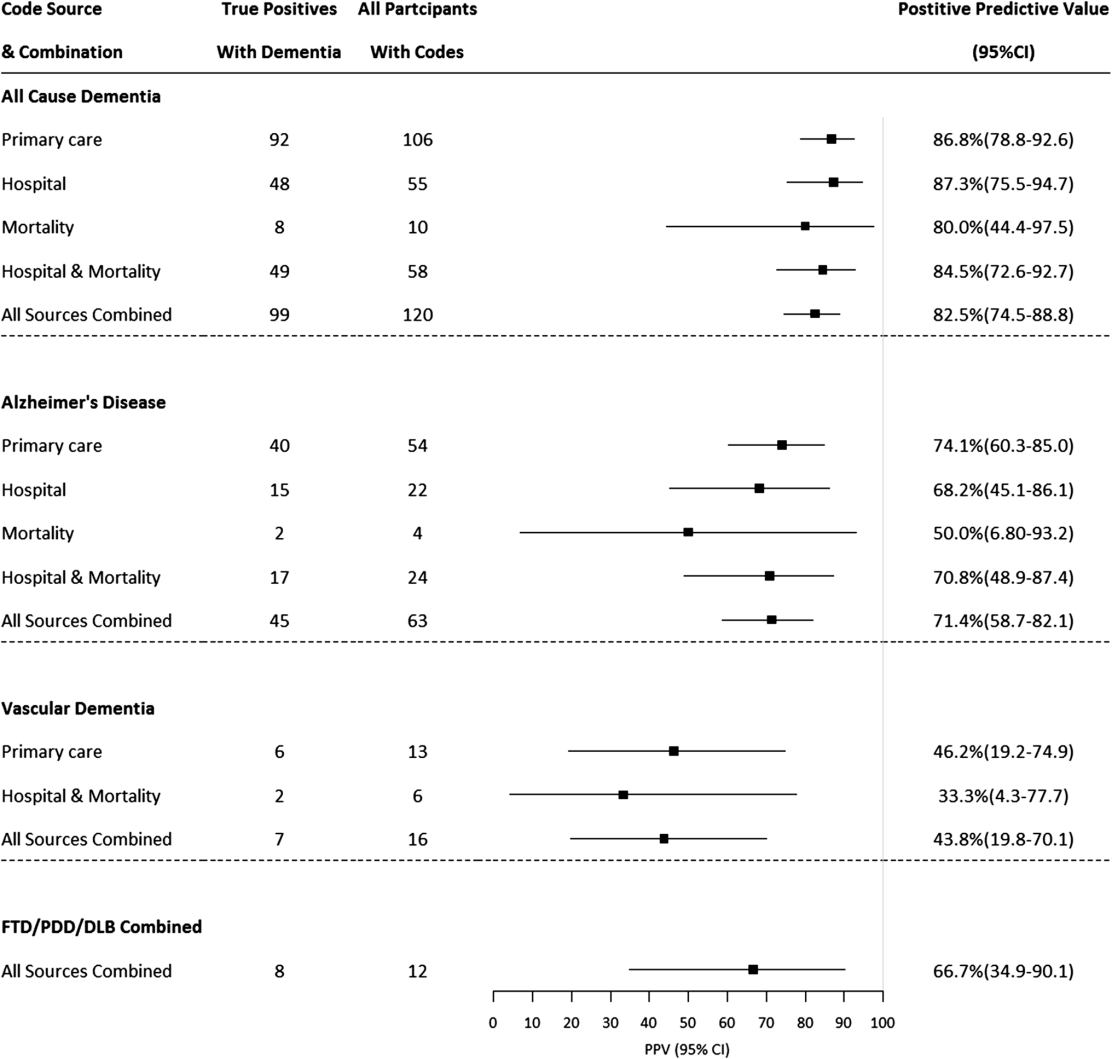
Positive predictive values for datasets, alone and in combination, stratified by dementia subtype. *FTD* frontotemporal dementia, *PDD* Parkinson’s disease dementia, *DLB* dementia with Lewy bodies. PPVs displayed for datasets where n ≥ 5. FTD, PDD and DLB combined into ‘other dementias’ category due to small numbers for each disease alone

For all-cause dementia, PPVs were 86.8% (95% CI 78.8–92.6), 87.3% (75.5–94.7) and 80.0% (44.4–97.5) in primary care, hospital admissions and mortality data respectively. PPVs were 84.5% (72.6–92.7) for hospital admissions and mortality data in combination, and 82.5% (74.5–88.8) across all datasets (Fig. 4). For subtype codes to identify those specific subtypes (e.g., Alzheimer’s disease codes identifying participants with Alzheimer’s disease), PPVs for all datasets combined were 71.4% (58.7–82.1) for Alzheimer’s disease and 43.8% (19.8–70.1) for vascular dementia.

### Sensitivity analysis: PPVs for all-cause dementia

When we removed from the analysis the six participants who had insufficient information in their medical record to confirm or refute a dementia diagnosis, PPVs for all-cause dementia increased to 88.5% (80.7–93.9) in primary care data, 92.3% (81.5–97.9) in hospital admissions, 88.9% (51.8–99.7) in mortality data, and 86.8% (79.2–92.4) across all datasets.

### Effects of additional criteria on PPV and case ascertainment for all-cause dementia

**Fig. 5:**
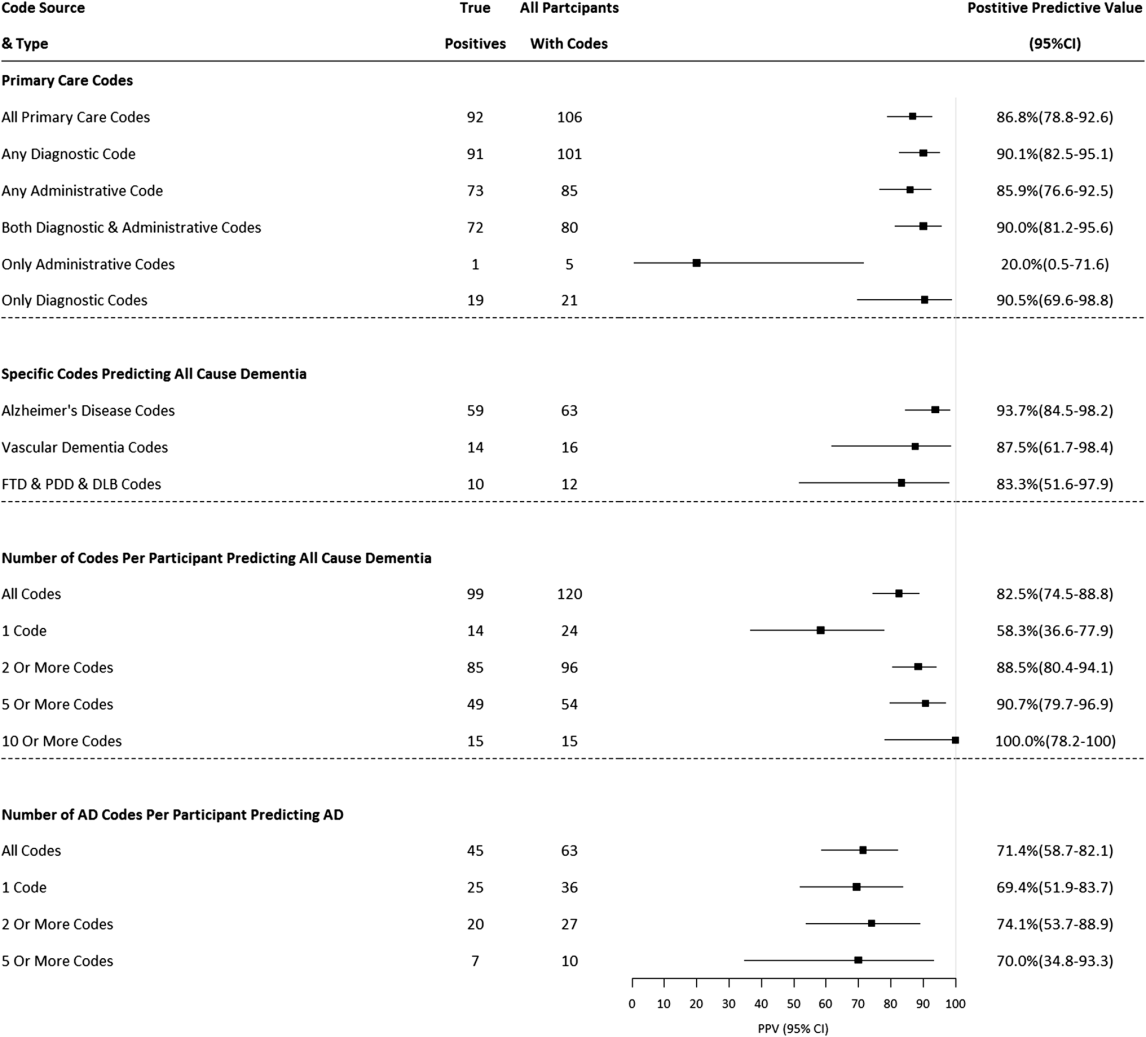
Effect of additional criteria on positive predictive values and numbers of cases ascertained. *FTD* Frontotemporal dementia, *PDD* Parkinson’s disease dementia, *DLB* dementia with Lewy bodies. ≥ 10 and ≥ 20 Alzheimer’s disease codes not shown due to small numbers (< 5). *Any Alzheimer’s disease, vascular dementia, FTD, PDD or DLB code to identify dementia of any cause

Figure 5 shows the effects of additional criteria on PPV and the number of cases identified. For primary care data, using only diagnostic codes (without administrative codes) appeared to increase PPV from 86.8% (diagnostic and administrative codes combined) to 90.1% without a large loss of cases (106 vs. 101, and only one of the lost cases was a true positive), although confidence intervals overlapped.

Using dementia subtype codes only to identify all-cause dementia resulted in an increase in PPV from 82.5 to 91.7%; however, only 84 cases were identified, compared to 120 using the broader code list (22/36 lost cases were true positive cases). The PPVs for Alzheimer’s disease, vascular dementia and the other specified subtype dementia codes to identify all-cause dementia were 93.7%, 87.5% and 83.3% respectively.

For all-cause dementia, PPV increased from 58.3% in participants with only one dementia code, to 88.5% for those with two or more codes, but with a reduction in the number of cases ascertained (120 vs. 96, true positive case numbers 99 vs. 85).

Table 2 summarises the PPVs and numbers of cases ascertained for three algorithms that appeared to balance a high PPV with good case ascertainment.

**Table 2:** Positive predictive value and case ascertainment in suggested algorithms to identify all-cause dementia cases in UK Biobank

Algorithm	Number of codes required	Dataset	PPV (95% CI)	Total (TP) cases identified
Any dementia code in any dataset	≥ 1 code in any dataset	P, H & M	82.5% (74.5–88.8)	120 (99)
Two or more dementia codes in any dataset	≥ 2 codes in any dataset	P, H & M	88.5% (80.4–94.1)	96 (85)
Any diagnostic code in primary care data*	≥ 1 diagnostic code	P	90.1% (92.5–95.1)	101 (92)

*P* Primary care, *H* hospital admissions, *M* mortality, *PPV* positive predictive value, *CI* confidence intervals. *TP* true positive

*Administrative read codes excluded

### Demographics of true and false positives

Demographic information for participants judged to be true positives and false positives are displayed in Table 3. False positive cases were less often female, had fewer codes, and were of lower average socioeconomic status.

**Table 3:** Demographics of participants who were adjudicated to be false positives, true positives and whole validation group

Group	Number of participants	Median age at recruitment (range)	Female (%)	Median age at first code (range)	Median number of codes (range)	Died during follow-up (%)	Median TDI (range)
All	120	67 years (43–70)	64 (53.3)	70 years (41–77)	5 (1–35)	25 (20.8)	1 (1–5)
True positives	99	67 years (51–70)	54 (54.5)	71 years (52–77)	6 (1–35)	20 (20.2)	1 (1–5)
False positives	21	67 years (43–70)	10 (47.6)	68 years (41–76)	2 (1–8)	5 (23.8)	3 (1–5)

*TDI* Townsend deprivation index—divided into quintiles (1—lowest deprivation, 5—highest deprivation) based on 2001 census data [18]

## Discussion

We have estimated the accuracy of using UK routinely-collected healthcare datasets, alone and in combination, to identify dementia cases, demonstrating PPV estimates of 80–87%. For subtype diagnoses, the PPV for identifying Alzheimer’s disease cases was lower than for all-cause dementia, but higher than that for vascular dementia (71% and 44% respectively across all datasets).

These PPV estimates are likely to be conservative, as we deemed potential dementia cases ‘false positives’ if there was insufficient information in the hospital medical record to confirm or refute a diagnosis of dementia. It is possible that some of these participants did have dementia, but relevant correspondence was missing. A sensitivity analysis, in which we excluded these participants from the PPV calculations, resulted in increased PPVs of 89–92% across the datasets. It is likely that the ‘true’ PPV lies between these conservative and less stringent estimates.

Acceptable levels of accuracy, and the relative importance of different accuracy metrics, depends on the context [19]. UKB is primarily used for research into the genetic and non-genetic determinants of disease [2]. In such analyses, where a sub-group within the cohort are identified based on their disease status, it is important to ensure that a high proportion of participants within the group truly do have the disease (high PPV) to minimise bias in effect estimates. A high specificity (the proportion of participants without the disease that do not receive a dementia code) is crucial in obtaining a high PPV, but is not in itself sufficient. In population-based prospective cohorts where dementia prevalence is low, the proportion of participants misclassified as having dementia (false positives) may be small (high specificity), even if the absolute numbers of false positives is high compared to the number of true positives (low PPV) [19]. Providing appropriate codes are used, the specificity of routinely collected healthcare data to identify disease cases in population-based studies is usually very high (98–100%) [20, 21]. For this reason, we designed our study to estimate the PPV of using routinely-collected healthcare data to identify dementia outcomes in UKB.

Primary care data is potentially a valuable resource for dementia case ascertainment. Our results show similar accuracy to hospital admissions and mortality data, in keeping with previous studies in this area [6, 7, 22]. Furthermore, 52% of cases were found only in primary care data, suggesting that using only hospital admissions and mortality data will miss cases. However, this finding is likely to be dependent on the age of the cohort, because as the cohort ages, more participants are likely to appear in hospital admissions and mortality data.

We explored the effect of various code selection criteria on PPV and the numbers of cases ascertained. The addition of primary care administrative codes added few extra true positive cases and reduced PPV. In keeping with previous findings [5], using specific dementia subtype codes to identify all-cause dementia and requiring ≥ 2 codes across any dataset led to higher PPVs but fewer cases identified. We identified three algorithms that, in this study, balanced a high PPV with reasonable case ascertainment. These algorithms include the use of primary care data, and to date, UKB has acquired linkage to primary care data for > 200,000 of its participants. These algorithms can, therefore, only be employed on the subset of the cohort in whom primary care data are available. An alternative approach would be to rely only on identifying cases within hospital admissions and mortality data for the whole cohort (> 500,000). In our study, this algorithm resulted in a PPV of 85%, but a reduction in case ascertainment from 120 to 58. Users of UKB data will need to select the approach that best suits their research question.

Sensitivity is another important accuracy metric to consider when comparing methods of identifying disease outcomes during follow-up in longitudinal studies. There is a trade-off between PPV and sensitivity, and any approach to identifying dementia cases must balance these in a way that is appropriate for the setting. Missing cases, and therefore a lower sensitivity, will reduce statistical power, but may also introduce bias if patients who are missed systematically differ from identified cases. We were unable to calculate the sensitivity of routinely-collected healthcare data to identify dementia outcomes in our study, because to do so the ‘true’ number of people with dementia in a population must be known, including those who have dementia but are currently undiagnosed, and therefore not known to healthcare services. UK mortality data has been shown to identify 45% of dementia cases, when diagnoses are taken from any position on the death certificate [21]. Sommerlad et al. [23] reported a sensitivity of 78% for hospital admissions data to identify dementia cases, using data from a large mental healthcare database as a gold standard. However, these patients were already known to mental health services with a diagnosis of dementia, so this does not account for people who were undiagnosed, meaning the true sensitivity is likely to be lower. The ongoing Cognitive Function and Ageing II Dementia Diagnosis Study is likely to provide the best estimate of the sensitivity of UK primary care data for identifying dementia diagnoses [24].

Our study has several strengths: creating a comprehensive code list; blinding of adjudicators to the coded information; using expert clinical adjudicators as the reference standard; allowing clinicians to make diagnoses mirroring current diagnostic practice, rather than relying on strict diagnostic criteria; and measuring intra-adjudicator agreement, showing it to be good for all-cause dementia.

There were some limitations, however. The UKB cohort is still relatively young, as indicated by the median age at first dementia code being 70 years, meaning our results may not be generalisable to settings with older populations. This is reinforced by the reference standard diagnoses, with a lower proportion of vascular dementia, mixed dementia and DLB cases than we would expect to see in older populations. Participants were all from a single centre in Scotland, and further research is necessary to ensure that our results are generalisable to other areas of the UK. Our sample size precluded in-depth analyses of vascular dementia and of other dementia subtypes such as DLB, PDD and FTD. The lack of a precise ICD-10 code for DLB means that we could only ascertain cases from primary care data. These are under-represented areas of epidemiological research using routinely-collected data, and a multi-centre study with longer follow up times will be necessary to accrue sufficient numbers. Lastly, our chosen reference standard is a potential limitation. We used correspondence and investigation results within the hospital EMR to adjudicate whether dementia was present. In some cases, the EMR may have been incomplete and there may have been additional information that would have been available to the clinician seeing the patient at the time of diagnosis. Our reference standard may therefore underestimate PPV by misclassifying some true dementia cases as false positives. Whereas inter-rater agreement was good for all-cause dementia, it was only moderate for subtype diagnoses. This is unsurprising, given that dementia subtype diagnoses lack objective diagnostic tests, and rely heavily on clinical judgement. It is well-recognised that many subtype diagnoses made in clinical practice do not agree with neuropathological data [25, 26], and so it is likely that our reference standard misclassified some diagnoses.

In conclusion, we have estimated the PPV of using UK routinely-collected healthcare datasets to identify cases of all-cause dementia, Alzheimer’s disease and vascular dementia during follow-up in large, prospective studies in the UK (specifically the UK Biobank resource) and have identified several algorithms that balance a high PPV with reasonable case ascertainment. Further research is required to investigate the potential biases inherent in using these data, the accuracy of coding in other dementia subtypes, and the generalisability of our findings to older ages and other geographical areas.

## Electronic supplementary material

Below is the link to the electronic supplementary material.

Supplementary material 1 (PDF 103 kb)

Supplementary material 2 (PDF 47 kb)

Diagnostic criteria used in adjudication (PDF 87 kb)
