# Supplementary material for: Identifying dementia outcomes in UK Biobank: a validation study of primary care, hospital admissions and mortality data
Source: Eur J Epidemiol. 2019 Feb 26;34(6):557–65. doi: 10.1007/s10654-019-00499-1 (PMC6497624; doi:10.1007/s10654-019-00499-1)
Supplement: Supplementary file 1 — Supplementary material 1 (PDF 103 kb) [file 10654_2019_499_MOESM1_ESM.pdf]

Electronic supplementary material 1 – Codes used to identify dementia cases and code selection process

Article: Identifying dementia outcomes in UK Biobank: a validation study of primary care, hospital admissions and mortality data

Authors: Tim Wilkinson, Kathryn Bush, Christian Schnier, Kristiina Rannikmäe, David Henshall, Chris Lerpiniere, Tom C Russ, Deborah Bathgate, Suvankar Pal, John T O'Brien, Cathie LM Sudlow on behalf of Dementias Platform UK

Corresponding author: Tim Wilkinson

Affiliations:

1. Centre for Medical Informatics, Usher Institute of Population Health Sciences and Informatics, University of Edinburgh , Edinburgh, UK
2. Centre for Clinical Brain Sciences, University of Edinburgh, Edinburgh, UK
3. Anne Rowling Regenerative Neurology Clinic, University of Edinburgh, Edinburgh, UK

Email: tim.wilkinson@ed.ac.uk

ICD-10

Description	Code	Subtype
Dementia in Alzheimer's disease	F00	AD
Dementia in Alzheimer's disease with early onset	F00.0	AD
Dementia in Alzheimer's disease with late onset	F00.1	AD
Dementia in Alzheimer's disease, atypical or mixed type	F00.2	AD
Dementia in Alzheimer's disease, unspecified	F00.9	AD
Alzheimer’s disease	G30	AD
Alzheimer’s disease with early onset	G30.0	AD
Alzheimer’s disease with late onset	G30.1	AD
Other Alzheimer's disease	G30.8	AD
Alzheimer's disease unspecified	G30.9	AD
Vascular dementia	F01	VD
Vascular dementia of acute onset	F01.0	VD
Multi-infarct dementia	F01.1	VD
Subcortical vascular dementia	F01.2	VD
Mixed cortical and sub-cortical vascular dementia	F01.3	VD
Other vascular dementia	F01.8	VD
Vascular dementia, unspecified	F01.9	VD
Binswanger's disease	I67.3	VD
Dementia in Picks disease	F02.0	FTD
Circumscribed brain atrophy	G31.0	FTD
Sporadic Creutzfeldt-Jakob disease	A81.0	O
Dementia in Creutzfeldt-Jacob disease	F02.1	O
Dementia in Huntington’s disease	F02.2	O
Dementia in Parkinson’s disease	F02.3	O
Dementia in HIV disease	F02.4	O
Mental and behavioural disorders due to use of alcohol - amnesic syndrome	F10.6	O
Dementia in other diseases classified elsewhere	F02	N
Dementia in other specified diseases classified elsewhere	F02.8	N
Unspecified dementia	F03	N
Delirium superimposed on dementia	F05.1	N
Senile degeneration of brain	G31.1	N
Other specified degenerative diseases of nervous system	G31.8	N

Read version 2

Description	Code	Subtype	Administrative or diagnostic code
[X] Dementia in Alzheimer's disease	Eu00.	AD	D
[X]Dementia in Alzheimer's disease with early onset	Eu000	AD	D
[X]Dementia in Alzheimer's disease with late onset	Eu001	AD	D
[X]Dementia in Alzheimer's disease, atypical or mixed type	Eu002	AD	D
[X]Dementia in Alzheimer's disease, unspecified	Eu00z	AD	D
Alzheimer’s disease	F110.	AD	D
Alzheimer’s disease with early onset	F1100	AD	D
Alzheimer’s disease with late onset	F1101	AD	D
Senile degeneration of brain	F112.	AD	D
[X] Other Alzheimer's disease	Fyu30	AD	D
Multi-infarct dementia	E004.	VD	D
Uncomplicated arteriosclerotic dementia	E0040	VD	D
Arteriosclerotic dementia with delirium	E0041	VD	D
Arteriosclerotic dementia with paranoia	E0042	VD	D
Arteriosclerotic dementia with depression	E0043	VD	D
Arteriosclerotic dementia NOS	E004z	VD	D
[X]Vascular dementia	Eu01.	VD	D
[X]Vascular dementia of acute onset	Eu010	VD	D
[X]Multi-infarct dementia	Eu011	VD	D
[X]Other vascular dementia	Eu01y	VD	D
[X]Vascular dementia, unspecified	Eu01z	VD	D
Cerebral degeneration due to cerebrovascular disease	F11x2	VD	D
Binswanger's disease	F21y2	VD	D
[X] Lewy body dementia	Eu025	DLB	D
Lewy body disease	F116.	DLB	D
[X] Dementia in Picks disease	Eu020	FTD	D
Pick's disease	F111.	FTD	D
Frontotemporal degeneration	F118.	FTD	D
Jakob-Creutzfeldt disease	A411.	O	D
Sporadic Creutzfeldt-Jakob disease	A4110	O	D
Alcoholic dementia, NOS	E012.	O	D
Dementia in conditions EC	E041.	O	D
[X] Dementia in other diseases classified elsewhere	Eu02.	O	D
[X] Dementia in Creutzfeldt-Jacob disease	Eu021	O	D
[X] Dementia in Huntington’s disease	Eu022	O	D
[X] Dementia in Parkinson’s disease	Eu023	O	D
[X] Dementia in HIV disease	Eu024	O	D
[X]Dementia in other specified diseases classified elsewhere	Eu02y	O	D
[X]Mental and behavioural disorders due to use of alcohol: amnesic syndrome	Eu106	O	D

[X]Mental and behavioural disorders due to use of alcohol: residual and late-onset psychotic disorder	Eu107	O	D
Cerebral degeneration due to Jacob-Creutzfeldt disease	F11x7	O	D
Cerebral degeneration due to Parkinson's disease	F11x9	O	D
Corticobasal degeneration	F11y2	O	D
H/O: dementia	1461.	N	A
Assessment of psychotic and behavioural symptoms of dementia	38C13	N	A
GDS level 4 - moderate cognitive decline	3AE3.	N	A
GDS level 5 - moderately severe cognitive decline	3AE4.	N	A
GDS level 6 - severe cognitive decline	3AE5.	N	A
GDS level 7 - very severe cognitive decline	3AE6.	N	A
Dementia monitoring	66h..	N	A
Dementia annual review	6AB..	N	A
Dementia medication review	8BM02	N	A
Shared care – prescribing drug for dementia	8BM50	N	A
Shared care – prescribing drug for dementia declined	8BM60	N	A
Antipsyc drug therapy dementia	8BPa.	N	A
Dementia advance care plan	8CMe0	N	A
Review of dementia advance care plan	8CMG2	N	A
Dementia care plan	8CMZ.	N	A
Dementia care plan agreed	8CMZ0	N	A
Dementia care plan reviewed	8CMZ1	N	A
Dementia care plan declined	8CMZ2	N	A
Dementia care plan review declined	8CMZ3	N	A
Dementia advance care plan agreed	8CSA.	N	A
Referral to dementia care advisor	8Hla.	N	A
Dementia adv care plan declnd	8IAe0	N	A
Dementia advance care plan review declined	8IAe2	N	A
Exception reporting: dementia quality indicators	9hD..	N	A
Excepted from dementia quality indicators: patient unsuitable	9hD0.	N	A
Excepted from dementia quality indicators: informed dissent	9hD1.	N	A
Dementia monitoring administration	9Ou..	N	A
Dementia monitoring first letter	9Ou1.	N	A
Dementia monitoring second letter	9Ou2.	N	A
Dementia monitoring third letter	9Ou3.	N	A
Dementia monitoring verbal invite	9Ou4.	N	A
Dementia monitoring telephone invite	9Ou5.	N	A
Senile and presenile organic psychotic condition	E00..	N	D
Uncomplicated senile dementia	E000.	N	D
Pre-senile dementia	E001.	N	D
Uncomplicated pre-senile dementia	E0010	N	D
Pre-senile dementia with delirium	E0011	N	D
Pre-senile dementia with paranoia	E0012	N	D

Pre-senile dementia with depression	E0013	N	D
Pre-senile dementia NOS	E001z	N	D
Senile dementia with depressive or paranoid features	E002.	N	D
Senile dementia with paranoia	E0020	N	D
Senile dementia with depression	E0021	N	D
Senile dementia with depressive or paranoid features NOS	E002z	N	D
Senile dementia with delirium	E003.	N	D
Drug induced dementia	E02y1	N	D
[X]Sub-cortical vascular dementia	Eu012	N	D
[X]Mixed cortical and sub-cortical vascular dementia	Eu013	N	D
[X] Unspecified dementia	Eu02z	N	D
[X] Delirium superimposed on dementia	Eu041	N	D

AD – Alzheimer's disease, VD – vascular dementia, FTD – frontotemporal dementia, DLB – Dementia with Lewy Bodies, O – other dementia subtype, N – no subtype specified.

D – diagnostic code, A – administrative code

Note:

In Scotland, hospital admissions data is available from two datasets depending on the type of hospital – admissions data from medical hospitals (SMR01) and psychiatric hospitals (SMR04). We combined SMR01 and SMR04 datasets as this is the format in which the data are available in England and Wales (Hospital Episode Statistics and Patient Episode Database for Wales respectively).

Code selection process

1. We manually searched ICD-10 (apps.who.int/classifications/icd10/browse/2010/en) and Read code browsers (isd.hscic.gov.uk/trud3/user/guest/group/0/pack/9) for relevant dementia codes.
2. We then compared our initial list with those used by studies identified in a previous systematic review[1], adding any additional codes we deemed likely to identify dementia with reasonable accuracy.
3. We scanned additional condition-specific databases for additional codes such as the UK Quality Outcomes Framework guidelines (content.digital.nhs.uk/qofbrv36).
4. We conducted a mapping exercise between ICD-10 and Read versions 2 and 3 (using data from isd.hscic.gov.uk/trud3/user/guest/group/0/home), adding any missing codes to create the final code list.

References

1. Wilkinson T, Ly A, Schnier C, et al. Identifying dementia cases with routinely collected health data: A systematic review. *Alzheimers Dement J Alzheimers Assoc*. April 2018. doi:10.1016/j.jalz.2018.02.016
