# Supplementary material for: Identifying dementia outcomes in UK Biobank: a validation study of primary care, hospital admissions and mortality data
Source: Eur J Epidemiol. 2019 Feb 26;34(6):557–65. doi: 10.1007/s10654-019-00499-1 (PMC6497624; doi:10.1007/s10654-019-00499-1)
Supplement: Supplementary file 2 — Supplementary material 2 (PDF 47 kb) [file 10654_2019_499_MOESM2_ESM.pdf]

Electronic supplementary material 2 – Adjudicator outcome form

Article: Identifying dementia outcomes in UK Biobank: a validation study of primary care, hospital admissions and mortality data

Authors: Tim Wilkinson, Kathryn Bush, Christian Schnier, Kristiina Rannikmäe, David Henshall, Chris Lerpiniere, Tom C Russ, Deborah Bathgate, Suvankar Pal, John T O'Brien, Cathie LM Sudlow on behalf of Dementias Platform UK

Corresponding author: Tim Wilkinson

Affiliations:

1. Centre for Medical Informatics, Usher Institute of Population Health Sciences and Informatics, University of Edinburgh , Edinburgh, UK
2. Centre for Clinical Brain Sciences, University of Edinburgh, Edinburgh, UK
3. Anne Rowling Regenerative Neurology Clinic, University of Edinburgh, Edinburgh, UK

Email: tim.wilkinson@ed.ac.uk

Appendix 2. Adjudicator outcome form

Adjudicator	
Participant identifier	

1. Sufficient information present to make a diagnosis of dementia (ICD-10 definition)

Yes	
Dementia likely, but formal definition not met using available information	
Not dementia	
Comments +/- alternative diagnosis (e.g. depression or mild cognitive impairment)	

2. If dementia present, subtype diagnosis (select one only)

	Meets formal criteria	Formal criteria not met, but subtype likely
Alzheimer's disease<br>(NIA-AA)		
Vascular dementia<br>(NINDS-AIREN)		
Mixed dementia<br>(meets NIA-AA criteria for AD and NINDS-AIREN criteria for VaD)		
Dementia with Lewy Bodies<br>(McKeith et al. 2005)		
Parkinson's disease dementia<br>(Emre et al. 2007)		
Frontotemporal dementia<br>bvFTD (FTDC) or PPA (Mesulam 2001)		
Other dementia<br>…………………………………………….		
No subtype diagnosis possible		

3. Any other comments ………...
