# Supplementary material for: Identifying dementia outcomes in UK Biobank: a validation study of primary care, hospital admissions and mortality data
Source: Eur J Epidemiol. 2019 Feb 26;34(6):557–65. doi: 10.1007/s10654-019-00499-1 (PMC6497624; doi:10.1007/s10654-019-00499-1)
Supplement: Supplementary file 3 — Diagnostic criteria used in adjudication (PDF 87 kb) [file 10654_2019_499_MOESM3_ESM.pdf]

**Electronic supplementary material 3 – Diagnostic criteria**

Article: Identifying dementia outcomes in UK Biobank: a validation study of primary care, hospital admissions and mortality data

Authors: Tim Wilkinson, Kathryn Bush, Christian Schnier, Kristiina Rannikmäe, David Henshall, Chris Lerpiniere, Tom C Russ, Deborah Bathgate, Suvankar Pal, John T O'Brien, Cathie LM Sudlow on behalf of Dementias Platform UK

**Corresponding author:** Tim Wilkinson

Affiliations:

1. Centre for Medical Informatics, Usher Institute of Population Health Sciences and Informatics, University of Edinburgh , Edinburgh, UK
2. Centre for Clinical Brain Sciences, University of Edinburgh, Edinburgh, UK
3. Anne Rowling Regenerative Neurology Clinic, University of Edinburgh, Edinburgh, UK

Email: tim.wilkinson@ed.ac.uk

## Appendix 3. Diagnostic criteria

### 1. Dementia – ICD-10 definition[1]

A syndrome due to disease of the brain, usually of a chronic or progressive nature in which there is disturbance of multiple higher cortical functions, including memory, thinking, orientation, comprehension, calculation, learning capacity, language, and judgement. Consciousness is not clouded. The impairments of cognitive function are commonly accompanied, and occasionally preceded, by deterioration in emotional control, social behaviour, or motivation.

### 2. Alzheimer's disease – NIA-AA criteria for probable AD dementia[2]

Meets criteria for dementia and in addition:
A. Insidious onset
B. Clear-cut history of worsening cognition
C. The initial and most prominent deficits are in one of the following categories:
- i) Amnestic – impairment in learning and recall
- ii) Non-amnestic – language, visuospatial or executive dysfunction

D. Diagnosis of probable AD should not be applied if there is:
- i) Substantial concomitant cerebrovascular disease
- ii) Features of Dementia with Lewy bodies
- iii) Prominent features of behavioural variant frontotemporal dementia
- iv) Prominent features of primary progressive aphasia
- v) Evidence of another cause that could have a substantial effect on cognition

### 3. Vascular dementia – NINDS-AIREN criteria[3]

Meets criteria for dementia and in addition:
A. Evidence of cerebrovascular disease
- i) Presence of focal signs on neurological examination
- ii) Evidence of cerebrovascular disease on brain imaging as evidenced by any or all of:
  - a. Large vessel infarcts
  - b. Single strategically placed infarct
  - c. Multiple basal ganglia and white matter lacunes
  - d. Extensive periventricular white matter lesions

B. A relationship between dementia and cerebrovascular disease as evidenced by one or more of:
- i) Onset of dementia within three months following a stroke
- ii) Abrupt deterioration in cognitive functions or stepwise progression of cognitive deficits

## 4. Dementia with Lewy Bodies – McKeith et al. (2005) criteria for probable DLB[4]

A. Central features (essential for a diagnosis):
   i) Dementia- progressive decline of sufficient magnitude to interfere with normal social or occupational function
   ii) Prominent or persistent memory impairment may not necessarily occur in the early stages but is usually evident with progression
   iii) Deficits on tests of attention, executive function and visuospatial ability may be especially prominent
B. Core features (either two or more core features required or one core feature accompanied with at least one suggestive feature from C.):
   i) Fluctuating cognition
   ii) Recurrent visual hallucinations that are typically well formed and detailed
   iii) Spontaneous features of parkinsonism
C. Suggestive features
   i) REM sleep behaviour disorder
   ii) Severe neuroleptic sensitivity
   iii) Low dopamine transporter uptake in basal ganglia demonstrated by SPECT or PET imaging
D. Supportive features
   i) Repeated falls and syncope
   ii) Transient, unexplained loss of consciousness
   iii) Severe autonomic dysfunction
   iv) Hallucinations in other modalities
   v) Systematised delusions
   vi) Depression
   vii) Relative preservation of medial temporal lobes on brain imaging
   viii) Generalised low uptake on SPECT/PET perfusion scan with reduced occipital activity
   ix) Abnormal (low uptake) MIBG myocardial scintigraphy
   x) Prominent slow wave activity on EEG with temporal lobe transient sharp waves
E. A diagnosis is less likely if:
   i) Presence of cerebrovascular disease evident as focal neurological signs or on brain imaging
   ii) Presence of any other physical illness or brain disorder sufficient to account for clinical picture
   iii) If parkinsonism only appears for the first time at a stage of severe dementia
F. Temporal sequence of symptoms:

   DLB should only be diagnosed when dementia occurs before or concurrently with parkinsonism (if present)

**5. Parkinson's disease dementia – Emre et al. 2007 for probable PD dementia**[5]

A. Core features (both must be present)

i) Diagnosis of Parkinson's disease
ii) A dementia syndrome with insidious onset and slow progression as defined by
    a. Impairment in more than one cognitive domain
    b. A decline from premorbid level
    c. Impairment in daily life, independent of the impairment ascribable to motor or autonomic symptoms

B. Associated clinical features

i) Typical profile of cognitive deficits including impairment in at least two of four core cognitive domains (attention, executive function, memory and language)
ii) Presence of at least one behavioural symptoms (apathy, depressed or anxious mood, hallucinations, delusions, excessive daytime sleepiness) supports the diagnosis, but lack of behavioural symptoms does not exclude the diagnosis

C. None of the following

i) Co-existence of any other abnormality which may by itself cause cognitive impairment
ii) Time interval between development of motor and cognitive symptoms not known
iii) Cognitive and behavioural symptoms appearing solely in the context of other conditions such as acute confusion due to systemic disease, drug intoxication or major depression
iv) Features compatible with vascular dementia according to NINDS-AIREN criteria

**6. Frontotemporal dementia – FTDC criteria for behavioural variant FTD (bvFTD)[6] and Mesulam criteria (2001) for primary progressive aphasia (PPA)[7]**

**FTDC criteria for possible bvFTD[6]**

1. Evidence of a neurodegenerative disease
    a. Progressive deterioration of behaviour and/or cognition
2. Three or more of the following
    a. Early behavioural disinhibition
    b. Early apathy or inertia
    c. Early loss of sympathy or empathy
    d. Early perseverative, stereotyped or compulsive/ritualistic behaviour
    e. Hyperorality and dietary changes
    f. Neuropsychological profile of executive/generation deficits with relative sparing of memory and visuospatial functions
3. Exclusion criteria (if (a) or (b) present than a diagnosis of bvFTD cannot be made. If (c) present then possible bvFTD can still be diagnosed but probably bvFTD cannot)
    a. Pattern of deficits is better accounted for by other disorder
    b. Behavioural disturbance is better accounted for by a psychiatric diagnosis
    c. Biomarkers strongly indicative of Alzheimer's disease or other neurodegenerative process

**Criteria for the diagnosis of PPA based on criteria by Mesulam 2001[7]**

1. Most prominent clinical feature is difficulty with language
2. These deficits are the principal cause of impaired daily living activities
3. Aphasia should be the most prominent deficit at symptom onset and for the initial phases of the disease

Exclusion criteria

1. Pattern of deficits is better accounted for by other disorder
2. Cognitive disturbance is better accounted for by a psychiatric diagnosis
3. Prominent initial episodic memory, visual memory and visuoperceptual impairment
4. Prominent, initial behaviour disturbance

**References**


1. World Health Organization. *The ICD-10 Classification of Mental and Behavioural Disorders: Clinical Descriptions and Diagnostic Guidelines*. Geneva: World Health Organization; 1992.

2. Jack CR, Albert M, Knopman DS, et al. Introduction to Revised Criteria for the Diagnosis of Alzheimer's Disease: National Institute on Aging and the Alzheimer Association Workgroups. *Alzheimers Dement J Alzheimers Assoc*. 2011;7(3):257-262. doi:10.1016/j.jalz.2011.03.004

3. Román GC, Tatemichi TK, Erkinjuntti T, et al. Vascular dementia Diagnostic criteria for research studies: Report of the NINDS-AIREN International Workshop*. *Neurology*. 1993;43(2):250-250. doi:10.1212/WNL.43.2.250

4. McKeith IG, Dickson DW, Lowe J, et al. Diagnosis and management of dementia with Lewy bodies: Third report of the DLB consortium. *Neurology*. 2005;65(12):1863-1872. doi:10.1212/01.wnl.0000187889.17253.b1

5. Emre M, Aarsland D, Brown R, et al. Clinical diagnostic criteria for dementia associated with Parkinson's disease. *Mov Disord*. 2007;22(12):1689-1707. doi:10.1002/mds.21507

6. Rascovsky K, Hodges JR, Knopman D, et al. Sensitivity of revised diagnostic criteria for the behavioural variant of frontotemporal dementia. *Brain J Neurol*. 2011;134(Pt 9):2456-2477. doi:10.1093/brain/awr179

7. Mesulam MM. Primary progressive aphasia. *Ann Neurol*. 2001;49(4):425-432.
